# Design of a Self-Measuring Device Based on Bioelectrical Impedance Analysis for Regular Monitoring of Rheumatoid Arthritis

**DOI:** 10.3390/s24082526

**Published:** 2024-04-15

**Authors:** JuYoung Jeong, Yun Soo Park, Eunchae Lee, SeoYoun Choi, Dokshin Lim, Jiho Kim

**Affiliations:** 1 Department of Mechanical and System Design Engineering, Hongik University, Seoul 04066, Republic of Korea; 2 Department of Industrial Design, Hongik University, Seoul 04066, Republic of Korea

**Keywords:** rheumatoid arthritis, inflammatory activity, bioelectrical impedance analysis, bioimpedance, self-measuring device, user-centered design, design engineering, heuristic evaluation

## Abstract

Rheumatoid arthritis (RA) is a chronic disease, in which permanent joint deformation is largely preventable with the timely introduction of appropriate treatment strategies. However, there is no consensus for patients with RA to monitor their progress and communicate it to the rheumatologist till the condition progresses to remission. In response to this unmet need, we proposed the design of a self-measuring device based on bioelectrical impedance analysis (BIA) for regular monitoring of inflammation levels. Twenty joints of both hands were measured to monitor trends in inflammation levels. Three electrodes were used to measure two joints of each finger. A central electrode was used for two consecutive measurements. A suitable form factor for the device was proposed for the vertical placement of the hand. To ensure the stability of measurements, an air cushion was incorporated into the back of the hand, hand containers were designed on both sides, and a mobile application was designed. We conducted a convergence-assessment experiment with five air pressures to validate the consistency and convergence of bioimpedance measurements. A heuristic evaluation of the usability around the product and mobile application was conducted in parallel by six subject matter experts and validated the design. This study underscores the significance of considering patients’ disease activity during intervals between hospital visits and introduces a novel approach to self-RA care.

## 1. Introduction

Rheumatoid arthritis (RA) is a chronic inflammatory autoimmune systemic disease that affects the synovial joints and leads to severe pain, swelling, and articular damage, with a global prevalence of approximately 1% [1,2,3]. In Korea, the most prevalent autoimmune rheumatic disease is seropositive RA. The prevalence of RA among the Korean population has increased slightly after the coronavirus disease and is estimated to reach approximately 1.39% by 2021 [4,5].

With the development of modern medical technology, joint deformation rarely occurs when it is accurately diagnosed and managed in the early stages of the disease [3,6,7,8]. However, without proper treatment, irreversible damage is caused to the joints within two years of onset, which results in permanent joint deformation [2]. The deformation of joints and resulting pain affect many aspects of the patient’s life and restrict their autonomy [9,10]. A study comparing joint damage in patients with RA who received early treatment and those who received delayed treatment showed that the erythrocyte sedimentation rate (ESR) and C-reactive protein (CRP) levels tended to be significantly lower in patients who underwent early treatment. In other words, early treatment significantly reduces disease activity and joint damage in patients with RA and lowers inflammation at the beginning of the disease, resulting in good treatment effects [11]. Patients with suspected RA are recommended to seek help within six weeks of symptom onset and undergo early management directed by a rheumatologist [12,13,14]. Consulting a rheumatologist within six weeks of symptom onset is beneficial for achieving sustained disease-modifying antirheumatic drug-free remission [14]. After three decades of focused efforts, scientists have now achieved the clinical application of the “treat to target” approach with initiation of aggressive therapy immediately after diagnosis and escalation of the therapy in pursuit of clinical remission in patients with RA [15]. However, despite the tremendous advances made over the past 20–30 years in the field of RA mechanisms, prognosis, and drug responses, current treatment modalities remain ineffective. No response to biological agents has been reported in 30–40% of patients [15]. Therefore, physicians must closely monitor the patient’s condition, understand the efficacy of medications, and choose the most suitable treatment for each individual [3,8].

It is critical for patients with RA to self-manage the disease in daily life while continuing their visits to a rheumatologist until remission is achieved. However, obstacles exist for timely and appropriate management. Here, we present a process for designing a self-measuring device that patients undergoing early treatment of RA can use daily at home.

## 2. Materials and Methods

**Figure 1:**
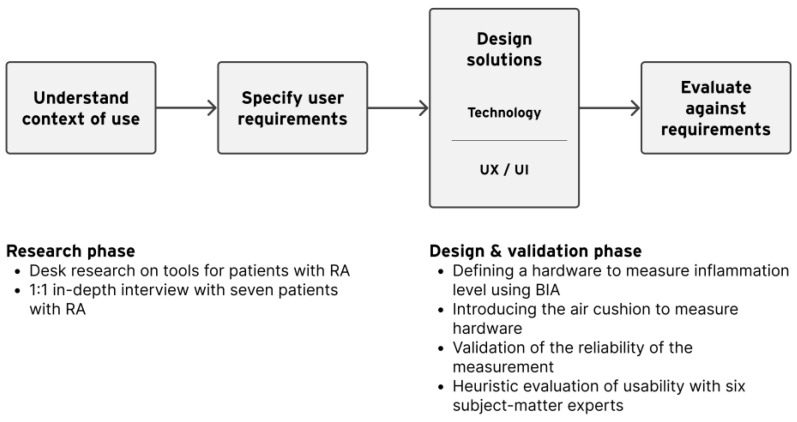
Our process of user-centered design.

User-centered design (UCD) is an iterative design process, in which designers focus on the users and their needs in each phase of the design process [16]. Our process of UCD involved users throughout the design process via a variety of research and design techniques, to create highly usable and accessible products for them (Figure 1). In the research phase, we conducted a series of studies to determine the real problems to be solved. By collecting various requirements, a functional description can be defined as the minimum viable product. In the design and validation phase, we examined the validity of the potential bioelectrical impedance analysis (BIA) technology. Finally, we requested subject-matter experts (SMEs) to heuristically evaluate the prototype from both feasibility and usability perspectives. At the end of the process, the design of a self-measuring device based on BIA for regular monitoring of RA was proposed.

### 2.1. Research

#### 2.1.1. Desk Research

RA tests assess disease activity during hospital treatment to determine the optimal treatment for each patient, even after diagnosis. This can improve the treatment rate and quality of life [17,18,19]. RA presents different symptoms and characteristics among patients; therefore, disease activity cannot be measured according to a single variable. Several methods are used to evaluate RA-disease activity. The Disease Activity Score of 28 joints (DAS28), Simplified Disease Activity Index, and Clinical Disease Activity Index are typically used [20]. Among them, DAS28, an international diagnostic guideline, is a method of summing the results of the three treatments and scoring them [21,22,23].

DAS28 incorporates physician palpation, blood inflammatory markers, and VAS (Visual Analog Scale). Physicians evaluate joint tenderness through physical examination, focusing on the 28 joints in the upper extremities primarily affected by RA [24]. Blood tests primarily confirm disease activity by measuring CRP levels, with rheumatoid factor (RF) and ESR checked if necessary [25,26,27,28]. The visual analog scale (VAS) represents the patient’s subjective perception of pain intensity and is used to assess conditions characterized by severe pain [29].

RA is diagnosed by comprehensively evaluating the results of various diagnostic tests. However, the most important physical examination requires considerable time for the process and also involves the examiner’s subjectivity and standards; therefore, the results may vary depending on the examiner [30].

Regardless of the improvement mentioned above, RA has a major impact on a patient’s activities of daily living [31]. To help patients with RA manage the disease activity by themselves, various tools and services are proposed. Henderson et al. [32] reviewed the state of the art of high-impact applications for wearable sensing devices used to measure an individual’s RA severity. Glove-type form factor offers advantages in measuring the range of motion (ROM), which is the main measure of joint stiffness. However, as measurement difficulty increases across patients, there remains significant ambiguity in patient’s diagnoses.

Another research proposed a photoacoustic imaging (PAI) technique for observing disease activity and bone erosion in RA by imaging synovitis [33]. However, the size of the measurement device is inevitably large, making it impractical for home use. Moreover, interpreting the data requires a high level of expertise.

In a separate research endeavor, an In Vitro Diagnostic (IVD) device for diagnosing RA was proposed [34]. Oh and Lee suggest a compact, user-friendly design, but the technology is invasive and meant only for medical use. The device is still too bulky for home use and has a medical device design, not suitable for home consumer electronics.

On the other hand, a cloud service was proposed to aggregate and provide diverse information about RA patients for treatment. It effectively addressed communication challenges between patients and doctors during treatment [35]. This might solve the problem of insufficient information during the intervals between hospital visits. However, the patient-uploaded Rheumatoid Arthritis Disease Activity Index (RADAI) to the cloud includes only subjective assessments of morning stiffness, joint range of activity scores, VAS, etc. [36].

Some mobile apps for RA offer features like education, lifestyle tracking, community connection, and self-monitoring [37]. Self-monitoring apps allow users to log daily symptoms, medications, and treatments. An ideal disease management app tracks activity, treatment, and behavior changes in line with self-management principles. While some apps let users upload clinical pictures for tracking physical changes in RA [38], most require manual input [39,40], leading to subjective recordings or hard-to-interpret data.

Our desk research to understand existing tools for patients with RA revealed that there are not yet any personal devices suitable for patients with RA to objectively monitor and grasp disease activity on their own. The existing hardware and services have limitations in terms of size (too big), usability (difficult to interpret the data), and design (not desirable look and feel).

#### 2.1.2. User Research

To understand in which context patients with RA manage the disease, we conducted 1:1 in-depth interviews with seven patients with RA. In Korea, the number of female patients is three times more than male patients [41]. Therefore, our interview focused more on empathizing with female patients. Interviewee information is presented in Table 1.

Patients with RA generally visit the hospital every 1–3 months. Of the patients who responded to the interview, three interviewees responded that they had difficulty in managing their health because of their inability to self-identify and define their disease activity, especially during the intervals between hospital visits. Furthermore, four of the patient-interview respondents commented on the specificity of RA symptoms. Participants answered that the areas of pain and symptoms were inconsistent and had changed. This is a characteristic of RA, and symptoms vary widely among patients, even within the same disease. Of the patients, six interviewees responded that they had difficulty communicating medical information with their doctors, even on the day they visited the hospital after the interval, and were dissatisfied with not receiving sufficient information as they wanted. They also stated that the treatment duration was short.

Summarizing the insights of the user research combining the opinions of patients and medical experts, the problems in the treatment of RA from patients’ perspectives were as follows:

- First, there are intervals between hospital visits; during that period, they were unable to self-identify and define their disease activity.
- Second, to make it even worse, the areas of pain and symptoms were inconsistent and had changed.
- Third, even on the day of visiting the hospital, they lack quality and amount of communication with the doctor so that their condition can be assessed accurately.

#### 2.1.3. User Requirements

Currently, the measurement of disease activity in RA is only possible when a patient visits the hospital. As mentioned earlier, the manual for measuring disease activity (DAS28) requires expertise. This is because medical experts and specialized devices are required for physical examinations and blood tests. Therefore, the patients’ disease-activity data are absent during the intervals between hospital visits. We found this to be an obstacle to the treatment of RA, making it challenging for physicians to provide effective treatment and for patients to manage their conditions effectively.

Consequently, our research focused on empowering patients to gather disease-activity data through self-measurement during intervals between hospital visits. To achieve this goal, we designed and developed a user-friendly self-measuring device. Therefore, we sought technology for devices that patients could utilize independently without the need for specialized expertise.

### 2.2. Design

#### 2.2.1. Sensor Technology

BIA, a non-invasive and extensively utilized technology, accurately identifies body components by measuring liquid levels through microcurrent [42]. Impedance indicates the degree of difficulty for electric current to flow in the living body and is inversely proportional to the moisture content in tissues [43,44]. When intra-articular inflammation occurs in comparison to normal joints, extracellular fluid levels increase, resulting in a reduction in bioimpedance. Therefore, we monitored changes in the degree of inflammation by measuring alterations in bioimpedance values.

Numerous studies have used BIA to quantify inflammation. BIA was used to detect inflammation in gums with periodontitis in a study [45]. Inflammation, characterized by an increase in fluid volume compared to normal tissue, is identified through bioimpedance methods. In this study, an experiment was conducted to measure the bioimpedance by attaching electrodes to normal gums and gums with periodontitis. Consequently, the bioimpedance of the inflamed area decreased by 35% compared to the bioimpedance of the normal gum area, whereas the bioimpedance of the peri-implantitis area showed a notable reduction of 56%. Another study examined a patient to evaluate the ankle joints using bioimpedance [46]. BIA was used to evaluate the degree of ankle swelling and damage to the ankle joint tissue.

It is evident, as in existing research, that the health status of tissues can be measured through bioimpedance. Therefore, we decided to employ BIA technology for the assessment of intra-articular inflammation levels with the aim of developing a self-measuring device utilizing this technology.

We devised an optimal assessment approach that aligns with current diagnostic methods for RA, considering the management characteristics of patients with chronic diseases. We selected 20 joints in both hands as the areas to be measured using the device. The current diagnostic method for RA is the DAS28, which evaluates 28 joints of the upper extremities, including 20 joints in both hands. In addition, RA preferentially invades small joints, such as the hands and feet [47].

**Figure 2:**
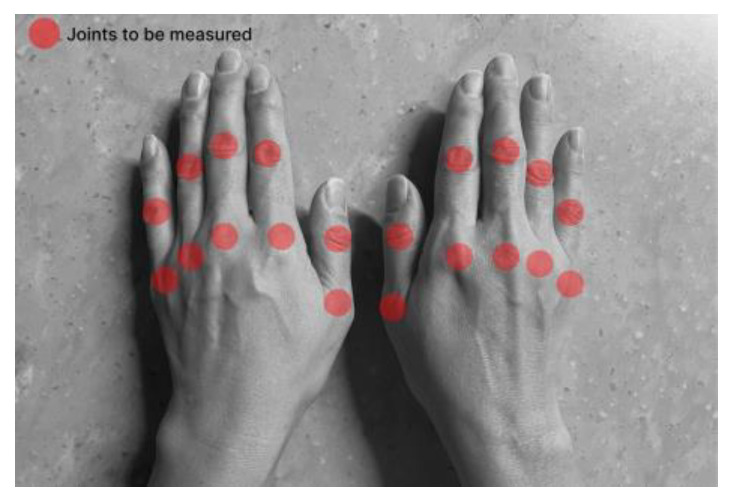
The location of the joints to be measured.

Based on a survey of patients with diabetes, a chronic condition, the primary challenge in self-managing the disease lies in maintaining consistent care practices [48]. To ensure that patients did not encounter difficulties in using the self-measuring device for consistent self-care, we opted to minimize the required patient actions when using the device. Therefore, the measurement site was determined to be 20 joints of the fingers as illustrated in Figure 2, excluding eight joints of the upper extremity. These joints were excluded because of their relatively low susceptibility to RA and the necessity for additional patient actions during measurement, given the considerable distance between each joint.

#### 2.2.2. Prototype Design

We considered various designs and mechanisms for manufacturing devices that target finger joints and improved the prototype several times through usability evaluation. The rationale for designing the prototype was as follows:

- Dimensions of hand insertion: The frame was designed to align with the average hand size of Korean women in their 50s, the age group with the highest incidence of RA, considering potential variations.
- Electrode-placement method: Thirty electrodes were positioned to enable the microcurrent to flow through the inflamed area.
- Protrusion of the electrode parts: The height of the hand frame portion near the electrodes was increased to enhance the adhesion between the electrodes and the hand.
- Hand-fixation method: An air cushion was used to ensure the stability of measurements by applying consistent pressure to the hand.
- Vertical insertion method for the hand: Because the device was designed for home use, we opted for a vertical insertion method to minimize volume.

First, the size of the hand-insertion section was crafted to fit the average hand size of Korean women in their 50s. According to statistics on Korean patients with RA, the prevalence rate in women is approximately three times higher than that in men, with women in their 50s comprising the largest proportion at 31.3% of the overall affected population [49]. Therefore, we referenced statistics on the body sizes of Koreans and tailored the hand-insertion section frame of the device to fit the average hand size of women in their 50s [50].

**Figure 3:**
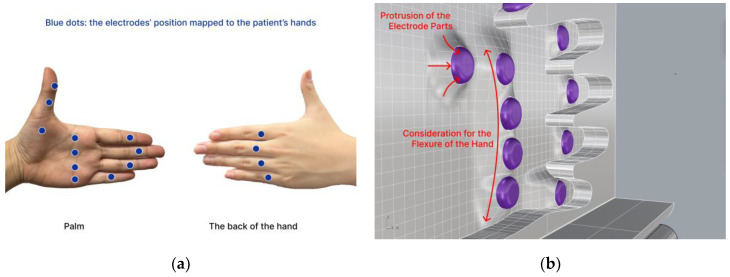
The electrodes are arranged as (**a**) the electrodes’ position mapped to the patient’s hands and (**b**) protrusion of the electrode parts, considering the flexure of the hand.

BIA was implemented by incorporating two electrodes per finger joint using the electrode on the back of each finger as a reference. The electrodes are arranged accordingly, as illustrated in Figure 3a,b. Biocompatible electrodes with Ag/AgCl were employed, featuring a planar surface with a circular diameter of 13 mm.

Rather than placing the electrodes side by side on the same surface, we arranged consecutive electrodes on opposite sides to allow the microcurrent to pass through the inflammatory area as it flowed between the electrodes. Therefore, as depicted in Figure 3a, two consecutive electrodes were positioned on the palm and back of the hand. Next, the electrode section protruded from the frame in contact with the hand, as shown in Figure 3b. This was performed to enhance the adherence between the electrodes and the hand, and a gradual elevation difference was implemented to ensure comfort for the hand.

During the measurements, it was essential that the hand remained still and stable. An air cushion on the back of the hand, as shown in Figure 3b, counterbalances the hand such that it remains in the right position. Finally, the form factor of our prototype defines the hand frame to be placed vertically so that the user feels comfortable while the device occupies as minimal space as possible.

**Figure 4:**
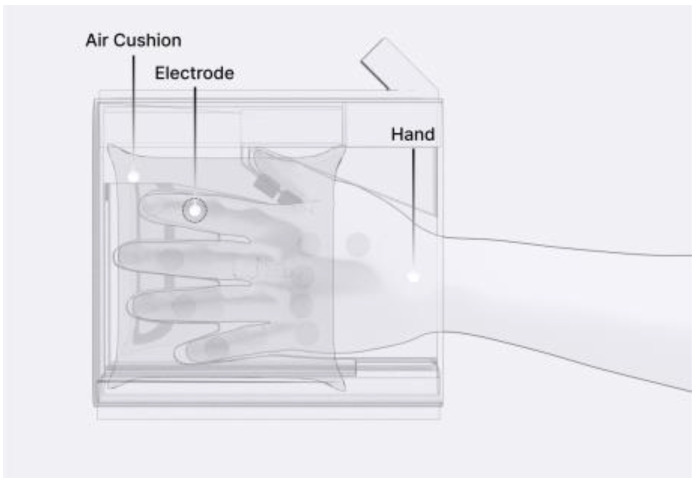
Prototype design transparent view.

The concept of an air cushion involves introducing controlled air pressure into the air cushion during the bioimpedance measurement phase to maintain consistent pressure between the user’s hand and the electrodes. An air-cushion mechanism was used to secure the user’s hand in the hand-insertion section, where the electrodes were positioned, to immobilize the measurement area. This also allows consistent pressure to be applied, ensuring proper contact between the skin and electrodes. A computer-aided design image illustrating this concept is shown in Figure 4.

#### 2.2.3. UX/UI Design

We decided to insert the hand vertically, and the air cushion was positioned vertically on the back of the hand to secure the hand during measurements. An air-cushion cover allows the air cushion inside to apply force and effectively immobilize the hand. The air cushion was designed to evenly apply pressure on the dorsal side of the hand. The air is pumped from the air pump and then enters the air cushion through an electronically controllable solenoid valve. The space between the two hand-insertion units accommodates various operational components. This includes a measurement circuit that receives bioimpedance information from the electrodes, a battery, two air pumps for both hands, two valves, and circuits controlling them along with the display.

**Figure 5:**
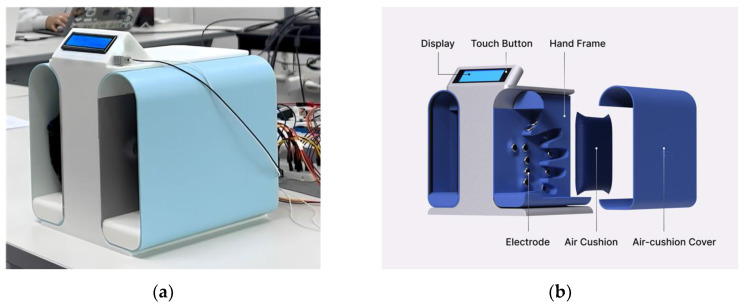
Prototype design: (**a**) prototype tested and (**b**) exploded view.

Patients can follow the measurement process by observing the device display, which provides information on the current measurement status, guidance for measurement, and display of the resulting data. The design of the prototype is illustrated in Figure 5.

**Figure 6:**
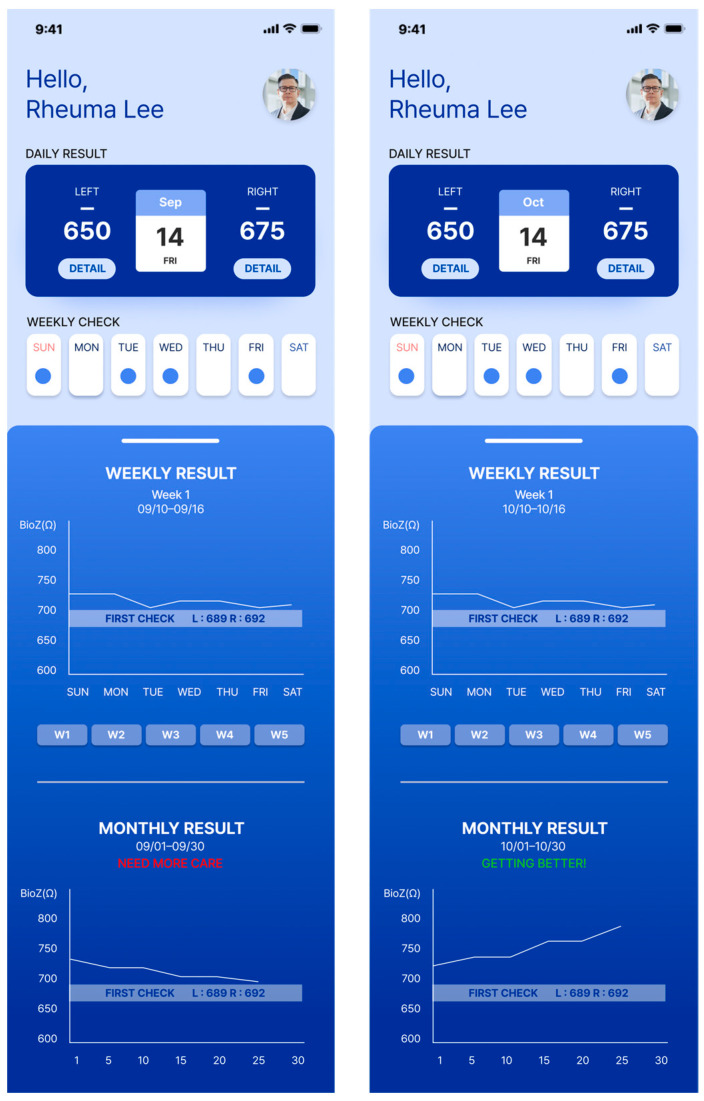
The patient’s screen example shown on the connected mobile application.

In addition, we designed a prototype mobile application to process and convey the measured disease activity to patients using this device. Upon entering the application, the average bioimpedance values for the left and right hands measured on the same day are displayed, as depicted in Figure 6. By pressing the detail button depicted for each hand, users can review the actual bioimpedance values for all 20 joints measured.

The device is important for accumulating daily bioimpedance data, thus showing a progression of disease activity for each patient. To facilitate patient tracking of the days on which they performed self-measurements, we introduced the “Weekly Check” section. A confirmation marker appeared on days when the measurements were conducted using the device within a week. On days without measurements, the space remained empty. Selecting a confirmation marker allowed users to review the bioimpedance values of both hands on a specific day.

The progression of disease activity (bioimpedance) as measured by the patient is shown in the form of a graph. Users can review the graphs in the “Weekly Result” and “Monthly Result” sections. Patients can engage in in-depth consultations with their healthcare providers during appointments armed with insights derived from these trends. The bioimpedance values decrease as inflammation occurs; therefore, a downward trend in the graph indicates a deterioration in the patient’s condition. Conversely, an upward trend indicated an improvement. By providing easily interpretable data, patients can play a proactive role in disease management.

## 3. Validation and Results

### 3.1. Technology Validation

To ensure the reliability of our technology in tracking changes in inflammation by measuring daily variations in bioimpedance, it is imperative that consistent bioimpedance values are observed at similar disease-activity levels. Therefore, experiments were conducted to verify the consistency and convergence of bioimpedance measurements. In BIA, the interaction pressure between the electrode and the skin significantly impacts the outcomes. Considering the concept of an air cushion involves air pressure controlling, experiments were conducted with multiple pressure options.

**Figure 7:**
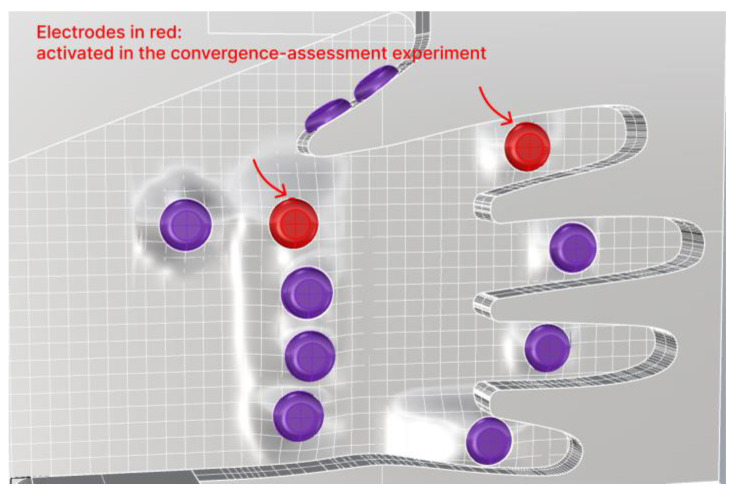
Two electrodes activated in the convergence-assessment experiment.

A male participant of the project in his 20s, clinically determined to be healthy, participated in our experiments. In the convergence-assessment experiment, we activated only two electrodes targeting the index finger, as illustrated in Figure 7, of the 20 joints on both hands that we aimed to measure.

**Figure 8:**
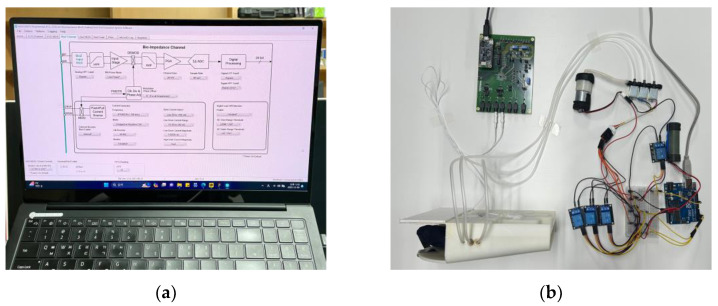
Setup for the convergence-assessment experiment: (**a**) computer settings for MAX30001G Evaluation Kit and (**b**) circuits and the hand-insertion unit configurations.

For the experimental setup, we connected the hand-insertion unit, including the air cushion, equipped with our electrodes, to the bioimpedance evaluation kit (MAX30001G Evaluation Kit, Analog Devices, Wilmington, MA, USA), which is designed for bioimpedance measurements. Data collection was performed using the software (MAX30001G ECG/BioZ AFE EVSYS Software, 3.1.2) provided by the same company. The experimental setup is illustrated in Figure 8.

BIA measures bioimpedance by passing a current through electrodes and requires the characteristics of the applied current to be determined. According to the International Electrotechnical Commission, the permissible value for current in contact with a patient for medical devices using electricity is 100 μA [51]. We set the maximum current applied to the participant at 8 μA, which is more than 100 times smaller than the typical threshold at which people generally begin to perceive electric current, which is 1 mA [52]. Biological tissues consist of cells and cell membranes that allow high-frequency currents to flow easily, whereas low-frequency currents face challenges in penetrating the tissues. Currents with frequencies below 50 kHz cannot pass through cell membranes and can only traverse the extracellular fluid. However, as the frequency increases, the current penetrates the cell membrane, allowing it to pass through both intracellular and extracellular fluids [43,53,54,55]. Our goal was to measure the level of inflammation in the joint synovium of patients with RA. Therefore, we determined a measurement frequency of 128 kHz, which is sufficient to detect results due to the interaction between intracellular and extracellular fluids. Thus, we selected a high drive current magnitude of 8 μA and frequency of 128 kHz.

**Figure 9:**
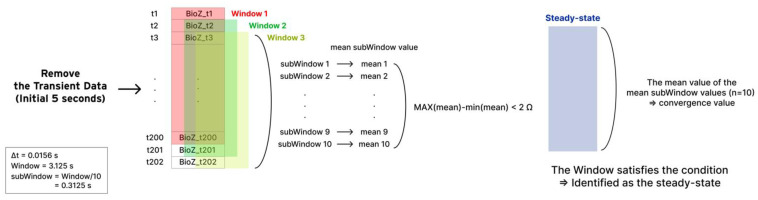
Schematic process for converging data.

The measurement of bioimpedance involved collecting bioimpedance [Ω] and data over time [s] using the MAX30001G Evaluation Kit, followed by preprocessing and analysis using MATLAB. At the beginning of the measurement, an initial transient state appeared with high bioimpedance values that gradually converged over time to the bioimpedance values in the localized region, where the two electrodes were placed. When the temporal changes in bioimpedance appear minimal variations, convergence is considered achieved. Hence, we defined the convergence criteria as the bioimpedance changes being sustained below 2 Ω for a duration of the “Window” (3.125 s). To reduce fluctuations within the Window, a “subWindow” approach was implemented, dividing the Window into 10 sections and taking the mean value for each subWindow. The schematic process for converging data is shown in Figure 9.

Explanation of the process for converging data is as follows:

- Remove the initial transient state of 5 s.
- Define 3.125 s of a continuous data bundle as a “Window”.
- In each Window, bioimpedance outliers are preprocessed. The data determined as outliers are replaced with the mean value of the corresponding Window.

The outlier equation is as follows:

Outliers = Bioimpedance < (mean − 2 × std) | Bioimpedance > (mean + 2 × std)(1)

- Define “subWindow” where the Window is divided into 10 sections. That is, each Window includes 10 subWindows.
- The steady state is identified when the difference between the maximum and minimum values of the mean subWindow values (*n* = 10) in the Window is less than 2 Ω.
- The convergence value is determined as the mean value of the mean subWindow values (*n* = 10).

**Figure 10:**
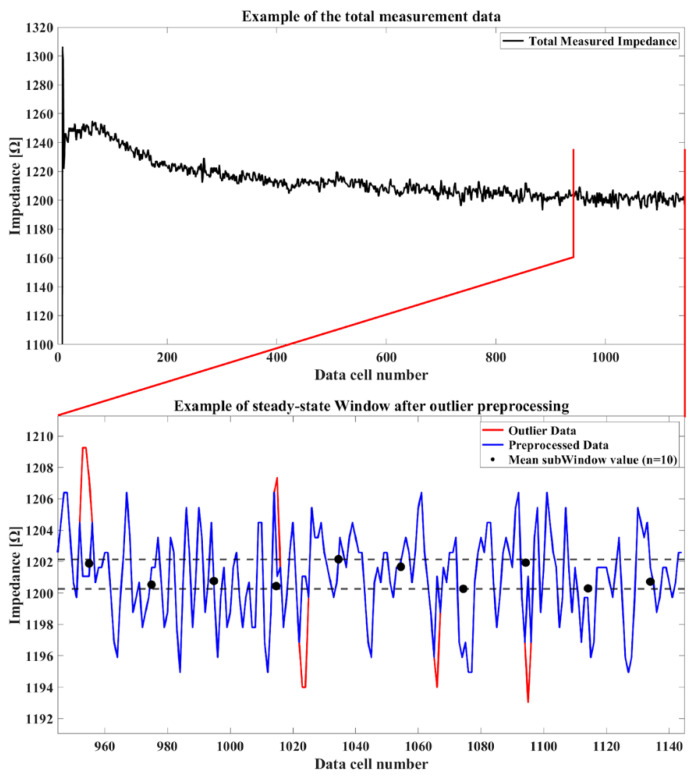
An example of the total measurement data and the steady-state Window.

An example of the total measurement data and the steady-state Window is shown in Figure 10.

After installing a load cell, an electro-mechanical sensor used to measure force, inside the hand-insertion unit, we applied five different air pressures to the air cushion. The force applied to the load cell is anticipated to closely resemble the force exerted on the back of the participant’s hand. The pressure transmitted from the air cushion to the participant’s hand was set to 8.8, 26.1, 45.7, 64.0, and 89.5 kPa for a total of five pressures, and the convergence value of bioimpedance was measured under identical conditions.

**Figure 11:**
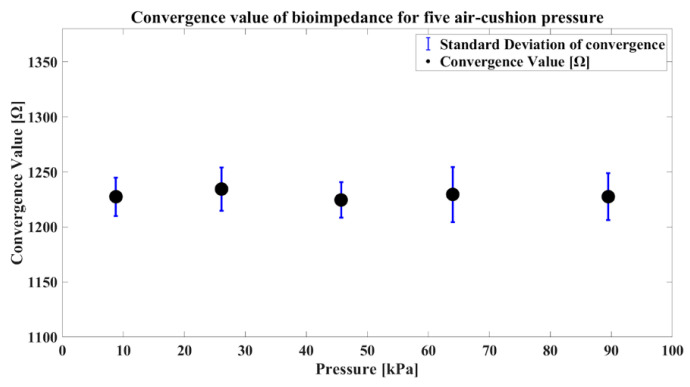
Convergence comparative graphs for each pressure level.

**Figure 12:**
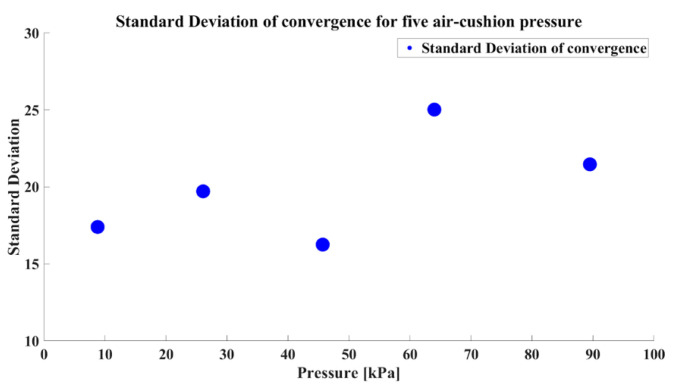
The standard deviation of convergence for each pressure level.

To minimize the direct impact of variations in internal and external body moisture on bioimpedance during the measurement process, all experiments were conducted in a single location with additional actions such as intermittently restricted food and water intake. The measurement site was cleaned with alcohol swabs before each session, dried in warm air, and the experiments were resumed. Bioimpedance was measured 20 times for each pressure level, and to prevent bias, experiments were conducted in a random order, with five repetitions for each pressure level. Given the identical measurement site with consistent disease-activity levels across the single participant, the median convergence value was comparable for each pressure level. Moreover, a lower standard deviation in the convergence value corresponding to a specific pressure indicates higher precision in measurements. The convergence value and standard deviation of convergence are shown in Figure 11 and Figure 12. As shown in Figure 11, the measurement is fairly repeatable since the standard deviation is less than 2% of the average for all the pressure conditions.

**Figure 13:**
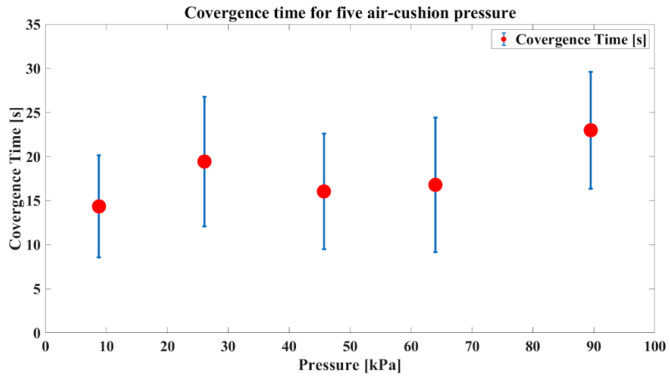
Convergence time comparative graphs for each pressure level.

Our self-measuring device was designed for regular use, making measurement time a crucial factor in terms of usability. Therefore, when selecting the air-cushion pressure, it is essential to consider not only the precision in measurements but also the time required to converge. The convergence times are presented in a single graph, as shown in Figure 13.

The results revealed that the convergence and convergence time were influenced by the air-cushion pressure. Additionally, considering our target population of patients with RA who need to avoid excessive pressure on their hands due to inflammation in the finger joints, we aimed to minimize the applied pressure. Therefore, we chose 45.7 kPa as the final air-cushion pressure considering the low standard deviation of convergence, short convergence times, and usability. We confirmed the need to optimize the air-cushion pressure through experiments with the prototype utilizing the air cushion. This experiment involved a single participant, and the determined optimal pressure may vary for different users. Hence, during the initial use of the device, it is advisable to measure the convergence in several pressure values for a specific user. Subsequently, the air-cushion pressure can be optimized based on convergence and convergence times to establish personalized and optimal usage settings for that user. This emphasized the importance of introducing an initial manual for the device.

### 3.2. Usability Evaluation

We conducted a usability evaluation of the prototype that incorporated the air-cushion method using heuristic evaluation. Heuristic evaluation [56,57] is a usability engineering method for finding the usability problems in a user interface design so that they can be attended to as part of an iterative design process. Heuristic evaluation involves having a small set of evaluators examine the interface and judge its compliance with recognized usability principles (the “heuristics”). The evaluation questionnaires were based on Jakob Nielsen’s heuristic evaluation criteria applicable to the device [58,59]. Among the recommended heuristics, three items were used: “Visibility of the System Status”, “Consistency and Standards”, and “Aesthetic and Minimalist Design”. We also introduced additional criteria related to the usefulness of the overall system and the usability of the air-cushion method. Furthermore, we referred to the System Usability Scale, a reliable method for evaluating usability, and formulated several assessment questions [60]. The questionnaire results are shown in Table 2.

The concept presented by Robert A. Virzi [61] regarding the saturation of problem discovery in usability testing (UT) suggests that as new participants are recruited in UT, the incremental contribution of additional participants to discovering new issues diminishes over time. In our case, recruiting six medical professionals and design consultants could potentially identify more than 75% of all the underlying usability issues [56]. Notably, when highly engaged users in the relevant field, so-called subject-matter experts (SMEs), participated, their responses saturated more rapidly. An SME has a high level of domain expertise and, by definition, is an expert in that area [62].

Table 3 outlines the careers and information of the SMEs who participated in our heuristic evaluation.

After explaining the concept of the device, each expert was evaluated independently by following our semi-structured process. Before responding to the questionnaire (in Table 2), we conducted interviews to gather opinions on the concept. The interview questions were as follows:

- What aspects do you consider crucial in the treatment of chronic diseases?
- To what extent do patients find it challenging to describe their condition during medical appointments?
- How much do you incorporate or reflect on these responses in your medical assessments?

A respondent emphasized the significance of “accessibility” in the treatment of chronic diseases. The respondent mentioned that not all patients have optimal accessibility to specialized medical professionals and suggested that our device could contribute to improving treatment accessibility for patients with limited access. All experts agreed that it was challenging for most patients to describe their condition during the intervals between hospital visits. They emphasized that the information that medical professionals can obtain about a patient’s condition during these intervals relies solely on the patient’s descriptions. Another respondent added that the format of patient responses varied, with some patients struggling to provide a description, while others provided excessively detailed explanations, such as daily pain diaries. Therefore, they expressed the belief that our device and the application’s trend data could assist in obtaining concise and comprehensive descriptions of a patient’s condition during short medical appointments.

**Figure 14:**
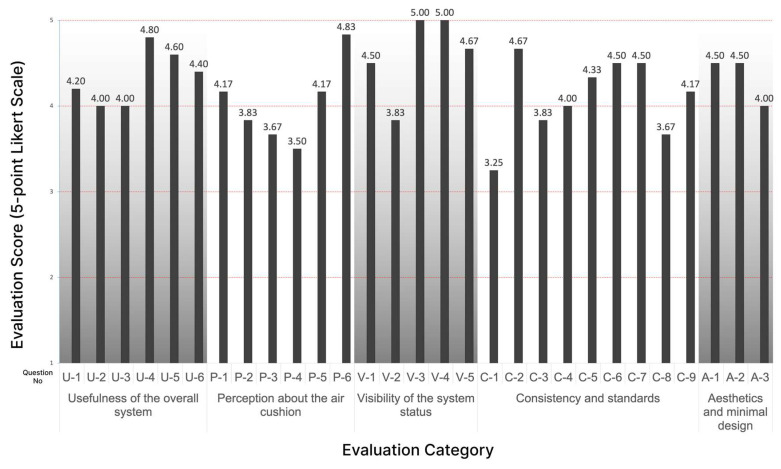
Result of the usability evaluation of the prototype (by questions).

**Figure 15:**
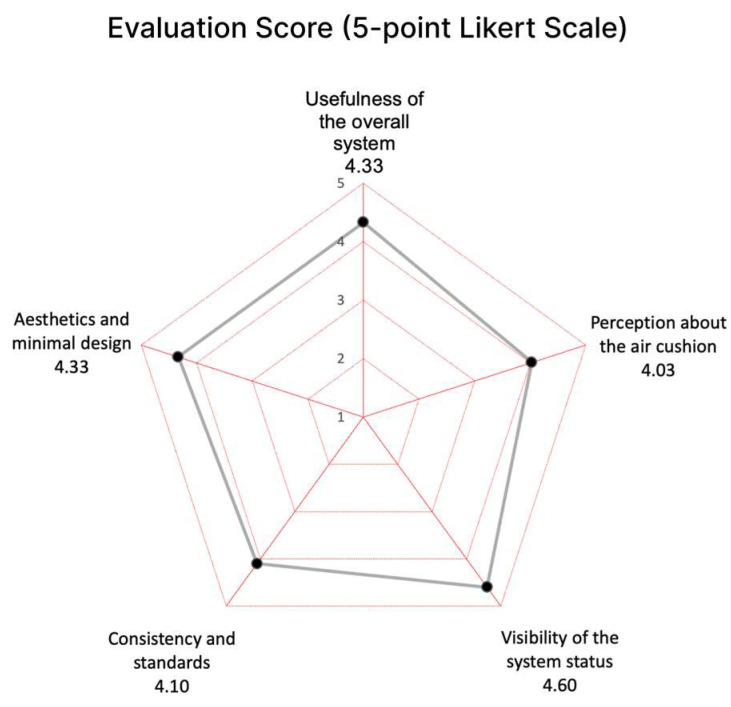
Result of the usability evaluation of the prototype (by categories).

The evaluation process commenced with the evaluator initiating responses to the usability evaluation questions in Table 2. The results are summarized in Figure 14 and Figure 15 and Table 4.

Experts generally gave positive evaluations of a mean of 4.33 (5-point Likert Scale) for the “Usefulness of the overall system” category. They confirmed that currently due to a lack of objective information on the patient’s disease activity during the visit interval, a medical staff had to rely only on the patient’s verbal explanation during the treatment. They said that it is important for the patient to know exactly about his or her condition in the management of chronic diseases. Therefore, they agreed that this lack of information is an obstacle to accurate treatment, prescription, and disease management of the patient. They stressed that it is particularly effective when the data are accumulated and delivered via mobile application as well as the device itself.

The “Perception about the air cushion” category resulted in the lowest mean of 4.03 (5-point Likert Scale) among all the categories. They agreed that the air-cushion method is effective and convenient in fixing the user’s hand during the measurement time. Since the hand can be fixed under the same pressure for each measurement, it will increase the precision of the measurements by enabling measurements under consistent conditions. However, most of them said that the air cushion around the palm and thumb does not adhere well enough to feel the fine touch to the electrodes.

The “Visibility of the system status” item received the highest evaluation of a mean of 4.60 (5-point Likert Scale). All of them clearly confirmed the discoverability and the ease of use of the device. It was highly evaluated that all the guidance on the device display was visible and clear, which is an essential quality of usability.

Regarding the “Consistency and Standards”, a few experts asked why only one hand was measured at a time. When they were explained that it was a decision for the user to be free of one hand to operate the button and for any possible emergency cases, they were immediately convinced. The rest of the experts said it would be more reasonable to measure only one hand at a time. Most of the experts answered that the disease activity information we represent could be refined further because currently, it is challenging for patients to comprehend the values of bioimpedance as we displayed directly from the measurements. They also mentioned that the average bioimpedance values of all joints of one hand would be meaningless. However, it was well received for showing the trend by accumulating disease activity weekly and monthly on the mobile application. Therefore, this category resulted in a respectively lower mean of 4.10 (5-point Likert Scale).

In terms of “Aesthetics and Minimal Design”, they evaluated the device’s design as aesthetic and enjoyable to use as a consumer device at home. They agreed to say that the design was reasonable to measure by inserting hands. There was a suggestion to make it more portable, not limited to one fixed place at home.

The usability of the air-cushion aspect yielded a lower score than the other usability aspects. Further research is required to identify specific user dissatisfaction points with the air-cushion feature and to enhance usability in those areas.

The consistency and standard categories displayed lower scores than other elements. The primary evaluation factor within this category was a question assessing the user’s comprehension of the application content. Difficulties were noted in the users’ intuitive understanding of numerical information, especially in the context of bioimpedance values. Subsequent improvements to the application prototype should consider user comprehension, necessitating research on providing numerical information in a more user-friendly manner, particularly for bioimpedance values. This underscores the importance of addressing these issues to enhance the usability in future iterations.

### 3.3. Final Design

**Figure 16:**
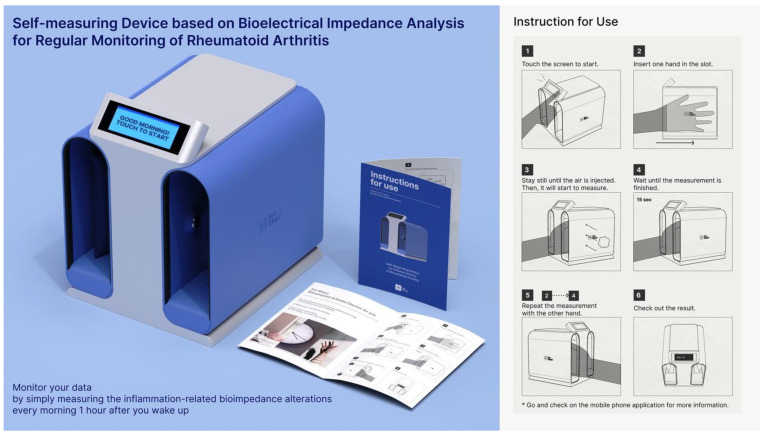
The final design and its instructions for use.

Finally, as illustrated in Figure 16, the vertical layout of the hand-insertion section and the container to secure the air cushion are tied to the BIA technology we employed. The process of using this product is simple as described in the instructions.

If the following behavior becomes the habit of patients with RA every morning after wake-up, their long-term treatment will be less ambiguous than now.

- Step 1: Touch the screen to start.
- Step 2: Put one hand in the hand-insertion section.
- Step 3: Stay still until the air is injected.
- Step 4: Wait until the measurement is finished.
- Step 5: Repeat from Step 2 to Step 4 with the other hand.
- Step 6: Check out the result on the small display of the device.

Notes: Go and check on the mobile phone application for more information. It is also recommended to set the alarm to regularly check an hour after waking up in the morning.

## 4. Discussion

Evidently, the use of this device is only limited to patients for whom joint deformations have not occurred yet. Also, patients with extreme sizes of hands would not get the right measurements due to the fixed position of electrodes. Further design research needs to search for a form factor to be free of the sizes of the hands and fingers.

Current data that we use on the user interfaces need to be further refined to meet the eye level of common users. Rather than directly using bioimpedance data, more user-friendly data or visualization of infographics can be considered. However, the mobile application itself cannot explain everything. Designing the ecosystem behind the device and the mobile application was not the scope of this paper, but ultimately, a related service should be connected. For example, the recommendation “NEED MORE CARE” or “GETTING BETTER” sentences could be used as a pathway command to connect to a remote doctor consultation service.

We also admit that the healthcare system in Korea may be very different from the ones in other countries. The time and effort that physicians could afford for each patient with RA seem also very limited, especially when the patient brings up new types of data. Nevertheless, through this study, we hope to draw attention to the importance of considering patient status during treatment hiatus periods in RA care, an aspect that has not been prominently addressed in previous studies.

The performance of our device relies on minimizing the standard deviation of convergence to enhance precision and ensure reproducibility in measurements. Therefore, further investigations are necessary to reduce this standard deviation. Specifically, since BIA is significantly influenced by the contact process between electrodes and skin, the selection of electrodes emerges as a critical variable in this technology. While a flat electrode was employed in this study, future research could concentrate on enhancing the stability of skin-to-electrode contact by incorporating a curved electrode shape.

Additional research involving experiments to monitor bioimpedance changes in RA patients with confirmed inflammation is essential. Validation of the device’s capacity to assess disease activity requires a comparison of bioimpedance changes of RA patients with the device’s standard deviation of convergence. Furthermore, aligning the trend in bioimpedance with hospital-assessed DAS28 provides rheumatologists with a diagnostic reference, thereby enhancing the potential for utilization.

## 5. Conclusions

In summary, patients can engage in more positive disease management through objective feedback, which enhances their self-efficacy. Physicians can make better diagnostic and prescription decisions by leveraging on a higher amount of data than that available before. The self-measuring technology utilized in this study, BIA, offers advantages in terms of usability such as short measurement times, non-invasiveness, and user-friendliness compared to conventional disease-activity measurement methods used in hospitals. To enhance the accuracy of disease-activity measurements using BIA, it is crucial to fix the measurement area at a consistent pressure for each measurement. Air cushions were implemented for accurate measurements, and through repetitive experiments, 45.7 kPa of air-cushion pressure which applied to the single participant was determined. The lowest standard deviation of convergence and short convergence times were observed around this pressure, indicating that precise measurements and thus may expect better usability. Additionally, as suggested in this study, the use of an application to automatically store measured values would be beneficial.

In this study, the experimentation on bioimpedance measurements focused on determining the device settings to ensure the convergence of measurements in localized areas. However, the limitations of bioimpedance changes owing to inflammation rely on the results of previous studies. Subsequent research is needed to confirm the ability of BIA to measure changes in inflammation within the finger joints, particularly in patients with RA. Nevertheless, this study is significant because it addresses the absence of patient status data during the treatment hiatus in the RA-treatment process, an aspect that has been overlooked in other studies.

In RA treatment, physicians must closely monitor the patient’s condition, understand the efficacy of medications, and choose the most suitable treatment for each individual [3,8]. In this regard, the RA self-measuring device proposed in this study could be considerably useful. Physicians can effectively prescribe medications by utilizing the abundant data accumulated by patients during medical intervals. Furthermore, the RA self-measuring device, which uses BIA and an air cushion as proposed by us, is expected to reduce the burden of measurement on patients, enabling them to continue management over an extended period. With our proposed BIA-based self-measuring device for RA-disease activity, patients can accumulate disease-activity data during intervals between hospital visits. By reviewing disease-activity data, patients can objectively assess their body condition, empowering them to play a proactive role in disease management.

## 6. Patents

This research work is based on three original patents pending invention by all authors of this paper: “An apparatus for self-measurement of disease activity of rheumatoid arthritis and a system comprising the same (10-2023-0190918)”; 26 December 2023; Republic of Korea, and “A medical device for home use (30-2023-0050189 and 30-2023-0050190)”; 8 December 2023; Republic of Korea.

## Figures and Tables

**Table 1:** Information of interviewed patients with RA (*n* = 7).

No	Sex	Age Group	Period of Managing RA
1	Female	40–49	2 years
2	Female	30–39	2 years
3	Female	60–69	20 years
4	Female	40–49	3 months
5	Female	40–49	10 months
6	Female	40–49	2 years
7	Female	40–49	3 months

**Table 2:** Questionnaires for heuristic evaluation.

Category	No	Question	
Usefulness of the overall system	U-1	The lack of information on the patient’s disease activity during the interval between the hospital visits makes the treatment difficult.	
	U-2	The patient’s statements about their disease are reliable.	
	U-3	Unclear statements about their symptoms and pain make it difficult to treat.	
	U-4	It is helpful for a rheumatologist to diagnose when patients know their disease activity.	
	U-5	It is helpful for a patient to know his/her disease activity.	
	U-6	Patients’ data on their disease activity, accumulated during the intervals between their visits, will be used for the diagnosis.	
	U-7	Patients’ data on their disease activity, available on the mobile app, will be used for better self-care of the patient’s health.	
Perception about the air cushion	P-1	The air-pressure method was suitable for fixing the hand inside during the measurement process.	
	P-2	The air pressure level was appropriate for fixing the hand.	
	P-3	During the measurement, there was no feeling of discomfort caused by the air pressure.	
	P-4	During the measurement, there was a feeling of close touch of my hand to the electrodes.	
	P-5	During the measurement, there was no feeling of frustration due to the lack of visibility of the positions to be measured on my hand inside.	
	P-6	When the measurement was completed, there was no feeling of discomfort caused by the air pressure.	
Visibility of the system status	V-1	It was easy to figure out the stages of progress (preparation, measuring, and completed).	
	V-2	It was cumbersome to press the button to move to the next step.	
	V-3	The message written on the LED screen (Put your right hand in and press the button) was clear.	
	V-4	The message written on the LED screen (Put your left hand in and press the button) was clear.	
	V-5	The message written on the LED screen (Please check on the mobile application for more information) was clear.	
Consistency and standards	C-1	It was not confusing to measure one hand followed by the other (not both hands simultaneously).	
	C-2	Using one button for dual function (power on and off and start the measurement) was not a problem.	
	C-3	The information shown on the screen of the mobile application regarding daily results was understandable.	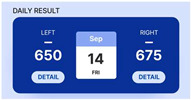
	C-4	The information shown on the screen of the mobile application regarding weekly results was understandable.	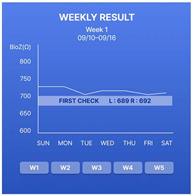
	C-5	The information shown on the screen of the mobile application regarding monthly results was understandable.	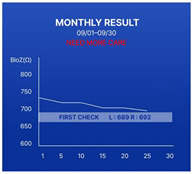
	C-6	The information shown on the screen of the mobile application regarding weekly checks was understandable.	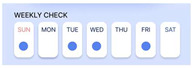
	C-7	The information shown on the screen of the mobile application regarding right-hand data was understandable.	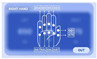
	C-8	The resulting value on the display was understandable.	
	C-9	It is easy to use intuitively and does not need any manual.	
Aesthetics and minimal design	A-1	The design can be well harmonized at home.	
	A-2	The size of the product is suitable.	
	A-3	The design well supports the physical measurement.	

**Table 3:** Information on participating SMEs (*n* = 6).

No	Job Title	Organization	Years of Expertise
1	Nurse	Severance Hospital	7 years
2	Nurse	Samsung Medical Center	10 years
3	Nurse	Samsung Medical Center	21 years
4	Nurse	Samsung Medical Center	28 years
5	Medical doctor (Rheumatology and Immunology)	Chung-Ang University Hospital	10 years
6	User researcher (Usability)	Embrain (a Macromill group)	25 Years

**Table 4:** Mean and standard deviation of the scores (*n* = 6).

Category	Mean	SD	Usability Issues
Usefulness of the overall system	4.33	0.606	Measuring and accumulating a patient’s disease activity during the intervals between hospital visits will certainly help doctors diagnose and patients manage the disease. It is effective to communicate this through the mobile app.
Perception about the air cushion	4.03	1.028	Fixing hands using air pressure is effective and convenient for users. In particular, the advantage is that it is fixed at the same pressure for each measurement. However, the palm and thumb felt less secure than other fingers.
Visibility of the system status	4.60	0.637	All the instructions displayed on the device display were well-visible and understandable.
Consistency and standards	4.10	1.067	The device’s design and usage are intuitive and easy to understand. However, some users could find it difficult to understand the value because it shows the bioimpedance value as a disease activity.
Aesthetics and minimal design	4.33	0.686	The device is aesthetically designed to be suitable for home medical devices. There was an opinion that it would be better if it was a more portable design.

## Data Availability

Data are contained within the article.
